# Phosphodiesterase 4 Inhibition in Neuropsychiatric Disorders Associated with Alzheimer’s Disease

**DOI:** 10.3390/cells14030164

**Published:** 2025-01-22

**Authors:** Jiming Chen, Zhengyao Zhu, Fu Xu, Baomin Dou, Zhutao Sheng, Ying Xu

**Affiliations:** 1 Department of Anesthesiology, Rutgers, The State University of New Jersey, Newark, NJ 07103, USA; cjming@126.com (J.C.); fuxu1784289008@163.com (F.X.); bd557@njms.rutgers.edu (B.D.); zs378@scarletmail.rutgers.edu (Z.S.); 2 School of Nursing and Rehabilitation, Nantong University, Nantong 226007, China; zzy206717293@163.com

**Keywords:** phosphodiesterase 4 (PDE4), cognitive deficits, Alzheimer’s disease (AD), major depressive disorder (MDD), anxiety, neuroinflammation

## Abstract

Cognitive disorders and psychiatric pathologies, particularly Alzheimer’s disease (AD) and Major depressive disorder (MDD), represent a considerable health burden, impacting millions of people in the United States and worldwide. Notably, comorbidities of MDD and anxiety are prevalent in the early stages of mild cognitive impairment (MCI), which is the preceding phase of Alzheimer’s disease and related dementia (ADRD). The symptoms of MDD and anxiety affect up to 80% of individuals in the advanced stages of the neurodegenerative conditions. Despite overlapping clinical manifestations, the pathogenesis of AD/ADRD and MDD remains inadequately elucidated. Until now, dozens of drugs for treating AD/ADRD have failed in clinical trials because they have not proven beneficial in reversing or preventing the progression of these neuropsychiatric indications. This underscores the need to identify new drug targets that could reverse neuropsychiatric symptoms and delay the progress of AD/ADRD. In this context, phosphodiesterase 4 (PDE4) arises as a primary enzyme in the modulation of cognition and mood disorders, particularly through its enzymatic action on cyclic adenosine monophosphate (cAMP) and its downstream anti-inflammatory pathways. Despite the considerable cognitive and antidepressant potential of PDE4 inhibitors, their translation into clinical practice is hampered by profound side effects. Recent studies have focused on the effects of PDE4 and its subtype-selective isoform inhibitors, aiming to delineate their precise mechanistic contributions to neuropsychiatric symptoms with greater specificity. This review aims to analyze the current advances regarding PDE4 inhibition—specifically the selective targeting of its isoforms and elucidate the therapeutic implications of enhanced cAMP signaling and the consequent anti-inflammatory responses in ameliorating the symptomatology associated with AD and ADRD.

## 1. Introduction

Neurocognitive disorders such as Alzheimer’s disease (AD) and major depressive disorder (MDD) represent a significant public health challenge, impacting millions of individuals in the United States and worldwide. According to the U.S. Department of Health and Human Services, memory deficits influence an estimated 55 million individuals globally, with AD accounting for 60% to 70% of these cases [1]. The continuum of AD has three phases: preclinical AD, mild cognitive impairment (MCI) due to AD, and dementia due to AD, i.e., AD and related dementia (ADRD) [2]. Neuropsychiatric manifestations, including depression and anxiety, are frequently observed in AD patients, beginning as early as the mild cognitive impairment (MCI) phase and affecting upwards of 80% of individuals in the advanced stages of AD [3,4]. AD and related dementia is a terminal stage of neurodegenerative disease that deteriorates memory and cognitive function, leading to the progressive decline of problem-solving ability, personality changes, and even death [3]. MDD is recognized as one of the most important symptoms of AD and ADRD worldwide, with an estimated 280 million individuals affected [4]. Although AD and MDD exhibit overlapping clinical features, the underlying pathogeneses remain to be clarified. Current pharmacological interventions aimed at preventing the progression of memory decline have limited efficacy [5] and cannot improve the triad of cognition, depression, and anxiety associated with AD. This circumstance prompts us to investigate the underlying mechanism and identify novel targets capable of mitigating cognitive and psychiatric symptoms, thereby preventing the progression of AD.

The hallmarks of AD are the progressive extracellular accumulation of the protein amyloid beta plaques (Aβ) and phosphorylation of tau that results in intraneuronal neurofibrillary tangles (NFTs) formation. Aside from Aβ and NFTs affecting neurons, the development of AD pathology includes astrogliosis and microglial activation, and the loss of neurons and synaptic components [5], all of which contribute to cortical atrophy. These findings are consistent with the pathological changes shared by AD patients. Notably, Aβ insults and Tau phosphorylation activate inflammatory responses by stimulating reactive oxygen species (ROS) and cytokine release, inhibiting secondary messenger-related signaling within the central nervous and peripheral systems (CNS and PNS), and ultimately resulting in AD and related dementia consequences [6,7]. Increasing evidence suggests that neuroinflammation plays a crucial role in developing and exacerbating AD/ADRD by downregulating second messenger levels, i.e., cAMP and cGMP in neurons and glial cells [8,9]. Phosphodiesterases (PDEs) have received widespread attention as potential targets for the treatment of neuropsychiatric indications. PDE is a large family and is divided into 11 family members, which control cAMP and cGMP levels in cells by degrading cAMP and/or cGMP, thus regulating the process of cell signal transduction. The inhibition of PDEs in the brain has emerged as an innovative strategy for treating AD/ADRD and related psychiatric disorders due to their ability to hydrolyze intracellular cGMP and/or cAMP. Among PDE families, phosphodiesterase 4 (PDE4) has been proven to be a competitive target of new drug development for AD/ADRD and MDD by regulating neuroinflammatory response [10,11,12]. This review summarizes the current research progress on the role of PDE4 and its subtypes in regulating cAMP signaling pathways and the inflammatory response in mediating symptoms of AD/ADRD and MDD, with a focus on investigating the crucial role of PDE4 in the pathogenesis of neuropsychiatric indications.

## 2. The Role of Neuroinflammation in AD/ADRD and MDD

### 2.1. Neuroinflammation in AD/ADRD

Emerging evidence suggests that neuroinflammation may serve as a pivotal pathological mechanism driving the progression of neurodegenerative disorders such as AD [13,14]. Microglia are resident immune cells in the brain [11]. In response to oxidative stress or brain injury, microglia become highly activated and release inflammatory cytokines such as IL-1α, IL-1β, and TNF-α, as well as free radicals and nitric oxide, triggering a pro-inflammatory cascade [13]. The accumulation of Aβ during the development of AD stimulates microglia and results in chronic neuroinflammation. The sustained release of inflammatory cytokines, in turn, increases amyloid-beta precursor protein (APP) and amyloid beta formation and decreases Aβ phagocytic receptors. Thus, the breakdown of amyloid plaques is reduced, which leads to the accumulation of Aβ plaques [15]. Some studies suggest that interleukin has the ability to enhance tau hyperphosphorylation by increasing the production of other cytokines, such as IL-16, and stimulating CDK5 activation [14]. This vicious cycle, i.e., Aβ/tau phosphorylation–neuroinflammation–more Aβ/tau phosphorylation–greater amounts of inflammatory cytokines cycle, exacerbates neurodegenerative progression, ultimately resulting in neuronal death and neuropsychiatric disorders such as AD/ADRD. Thus, neuroinflammation is one of the major mechanisms in AD pathology, and the activated glial and microglial cells are key players in neuroinflammation. Consistent with this statement, some clinical studies have demonstrated that cytokines, such as IL-1α and IL-1β, are elevated in the serum of patients with AD and mild cognitive impairment patients, indicating the critical roles of these increased cytokines in neurodegeneration [16]. Systemic inflammatory responses are accelerated in the progression of AD, further worsening cognitive decline and delirium symptoms [17]. Furthermore, aging is another important risk factor for neuroinflammation in these diseases. As individuals age, the blood–brain barrier (BBB) becomes more permeable to immune cells and chemicals [13], and the risk of AD may double in the setting of systemic infection [18]. Studies have shown that inflammatory substances such as C-reactive protein and IL-6 are elevated in plasma several years before the clinical onset of cognitive impairment compared to people of a similar age but without cognitive problems [18,19]. Genome-wide association studies revealed genes associated with increased AD risk, including TREM2, CLU, CR1, EPHA1, ABCA7, MS4A4A/MS4A6E, CD33, and CD2AP, which regulate Aβ clearance and participate in immune response and inflammation. This finding suggests that neuroinflammation is a critical factor in disease progression during both the early and late stages of AD [20].

In addition to microglial involvement, recent evidence implicates astrocytes in the etiopathogenesis of AD. Astrocytes, traditionally recognized for their neuroprotective functions, are integral to maintaining central nervous system homeostasis. They facilitate neurotransmitter modulation, trophic factor release, synaptic plasticity, oxidative stress mitigation, and the safeguarding and specialization of dopaminergic neurons [21]. The activation of astrocytes in response to neuronal injury prompts the release of pro-inflammatory cytokines and triggers a series of cascades characterized by oxidative stress, demyelination, and neuronal apoptosis. Concomitantly, astrocytes manifest a reactive phenotype, encircling amyloid deposits and contributing to glial scar formation via the phagocytosis of damaged dendrites and synaptic elements in the development of AD. However, reactive astrocytes are also implicated in the facilitation of Aβ clearance, and their activity has been posited to exert neuroprotective effects that may outweigh their contribution to neurodegeneration [22]. Although the role of astrocytes in AD remains a subject of scientific debate, further research is necessary to elucidate their exact role in disease onset and progression, particularly in the context of neuroinflammation. Current therapeutic strategies in AD research are increasingly targeting CNS inflammation inhibition [23]. At present, there are no unique inflammatory biomarkers for diagnosing or monitoring AD [23,24]. Neuroinflammation is recognized not as a solitary causative factor but rather as a consequential pathology that would intensify the severity of AD/ADRD.

### 2.2. Neuroinflammation in MDD

Current neurobiological frameworks for the pathophysiology of MDD encompass several substantive hypotheses. The monoamine hypothesis proposes that depression is caused by the monoamines’ alteration, including serotonin, norepinephrine, and dopamine [24]. Complementing this, the stress-related theory extends the monoamine hypothesis that demonstrates that the dysregulation of the hypothalamus–pituitary–adrenal gland (HPA) axis is the key factor, thereby contributing to the pathophysiological profile of depression [25]. A recent study proposed the brain-derived neurotrophic factor (BDNF) hypothesis, which demonstrates that depression is the consequence of attenuated neurogenesis and diminished neuroplasticity, with sequelae attributed to reduced levels of BDNF. Parallel to these established paradigms, emerging data support an inflammatory hypothesis of depression, postulating that an upsurge in pro-inflammatory cytokines is a contributing factor. Several meta-analyses have revealed increased levels of inflammatory cytokines, such as IL-1β, soluble IL-2, IL-6, TNF-α, C-reactive protein (CRP), and inflammatory mediator PGE2, in the peripheral blood and cerebrospinal fluid (CSF) of patients with the major depressive disorder [26,27,28,29]. Research has provided evidence suggesting an inverse relationship between prior antidepressant treatment and levels of inflammatory markers, with lower levels observed post-treatment [30]. Furthermore, a disparity in the plasma concentrations of IL-6 and acute-phase reactants has been documented between patients with varying responses to antidepressant therapy, indicating higher levels in those with reduced therapeutic responsiveness [31]. It is still uncertain whether these inflammatory cytokines affect the central nervous system function by activating inflammatory pathways in the brain or crossing the BBB from the periphery. Nevertheless, it is recognized that such cytokines can penetrate the CNS and interact with critical processes implicated in the pathogenesis of depression. These include alterations in neurotransmitter metabolism, the disruption of neuroendocrine function, and the impairment of neural plasticity [30].

Notably, cytokines within the CNS have been shown to modulate the synthesis, release, and reuptake of neurotransmitters that are pivotal in the context of depression, specifically serotonin, norepinephrine, and dopamine. In addition, the activation of peripheral inflammatory signals by the activation of an array of intracellular signaling, including the signal transducer and activator of transcription 1a (STAT1a), interferon regulatory factor-1 (IFR-1), p38 mitogen-activated protein kinase (MAPK), and nuclear factor-κB (NF-κB), may activate the enzyme indoleamine 2,3 dioxygenase (IDO), thereby modulating the tryptophan metabolism pathway, affect the synthesis and utilization of the monoamine transmitters [32]. The activation of p38 MAPK pathways has been linked to the induction of reuptake transporters for neurotransmitters such as serotonin, dopamine, and norepinephrine [33,34]. The activation of NF-κB, p38 MAPK, STAT5, and cyclooxygenase pathways can also inhibit glucocorticoid receptor (GR) function by disrupting GR translocation and inhibiting GR-DNA binding. Such a disruption of glucocorticoid signaling may contribute to the hyperactivity of the corticotropin-releasing hormone and promote more inflammatory reactions by negative feedback regulation of the HPA axis [34]. Considering the impact of neuroinflammation on the progression of depression-like behavior, many studies have explored therapeutics for MDD using non-steroidal anti-inflammatory agents, cytokine inhibitors, and monoclonal antibodies against specific cytokines. These approaches have demonstrated effectiveness in alleviating depressive symptoms, with only a relatively low-to-moderate side effect [35,36]. Consequently, the modulation of neuroinflammatory processes presents an alternative therapeutic strategy in the treatment arsenal for MDD.

## 3. The Role of PDE4 and Its Selective Inhibitors in AD and MDD

The activation of transcription factors such as the cAMP-response element-binding protein (CREB) in the brain causes the gene transcription required to consolidate learning and memory [37]. PDEs have 11 gene families, i.e., PDE1-PDE11, primarily based on their sequence homology. These subtypes are classified into three main categories of substrates: cAMP-specific PDEs (PDE4, PDE7, PDE8), cGMP-specific PDEs (PDE5, PDE6, PDE9), and those targeting both cAMP- and cGMP-PDEs, such as PDE1, PDE2, PDE3, PDE10, and PDE11 [37,38]. Out of these substrates, PDE1, PDE2, PDE4, PDE9, and PDE10 are highly expressed in the cerebral cortex, hippocampus, amygdala, striatum, hypothalamus, olfactory system, and adrenal gland. These distribution patterns suggest an association with oxidative stress-related depression, anxiety, and learning and memory, as well as schizophrenia, stroke, alcohol consumption, and drug abuse. The inhibition of these PDEs could hypothetically increase cyclic nucleotides in the endothelial cells of brain vessels and neurons; with the increased levels of cAMP and cGMP, the activation of second messenger-related signaling pathways could decrease inflammation and promote neuroprotection [38]. Due to the essential role of cellular signaling of cyclic nucleotides and the rich expression of PDEs in the CNS, there are indications that cAMP, cGMP, and the inhibition of PDEs regulate neuronal cell survival. CREB is activated by multiple different kinases, including PKA and MAPK, which are associated with the upregulation of neurotransmitters, growth factors, and other signaling molecules important for neuroplasticity and neuronal survival. The brain-derived neurotrophic factor (BDNF) gene contains a cAMP response element (CRE) that serves as a binding site for phosphorylated CREB, which subsequently stimulates transcription [39]. Once CREB is activated, it regulates neuronal survival and growth, as well as memory formation [40]. With the inhibition of PDEs, the resulting elevation in cyclic nucleotides, i.e., cAMP and/or cGMP, would play an essential role in neuroprotective effects. PDE4 is one of the subtypes that controls intracellular cAMP concentrations inside the CNS by hydrolyzing cAMP in neurons. PDE4 inhibition causes a cAMP build-up in the brain, which activates downstream signaling, such as PKA and CREB phosphorylation [41]. The AC/cAMP/PKA/CREB signaling pathway plays a role in memory acquisition, consolidation, and retrieval, as well as in antidepressant action and other central functions. This pathway is downregulated in patients with AD [42]. PDE4 expression is usually highly expressed in AD patients [43]. In contrast, PDE4 levels are also much lower in MDD patients than in AD patients, which is thought to be a compensatory strategy in response to decreased cAMP signaling produced by impaired neurotransmission [44]. As a result, PDE4 would be a major player in mediating cognitive function and memory performance.

### 3.1. PDE4 and Its Selective Inhibitors in AD

The cAMP/PKA/CREB signaling cascade is an essential pathway for memory consolidation and reconsolidation and is also integral in activating the gene expression necessary for the establishment of long-term memory across various animal models [39]. Rolipram was the first selective PDE4 inhibitor to be investigated in the early 1990s for the treatment of depression. Despite its subsequent discontinuation due to a limited therapeutic window and a high incidence of adverse effects, including nausea and emesis, it still provided the foundation for further research into and the development of other PDE4 inhibitors currently undergoing clinical trials [45]. Several studies have demonstrated that rolipram ameliorates or delays disease progression by increasing the cAMP levels in the brain in APP/PS1 transgenic mouse models [45,46] and reverses acute changes in dendritic spines caused by the Aβ peptide [47]. Furthermore, evidence indicates that in the CA1 region of the hippocampus [46], rolipram reverses the Aβ42-induced impairment of long-term potentiation (LTP), one of the cellular mechanisms considered important in learning and memory formation. The fact that PDE4 inhibition effectively reverses memory deficits has further been corroborated by a series of studies [48,49]. Preclinical studies have demonstrated that rolipram alleviates memory deficits and depression-like behavior by reducing the amyloid-β burden, tau phosphorylation, neuroinflammation, apoptosis, and neuron loss via the activation of cAMP/PKA and cAMP/EPAC/ERK signaling pathways [50]; roflumilast (another PDE4 inhibitor) leads to the accumulation of cAMP and subsequent increases in pCREB, BDNF, and the ratio of Bcl2/Bax in the brain, which can improve learning and memory function and attenuate depression-like behavior in AD mice [51]. Roflumilast increases cAMP, p-CREB, and BDNF levels, reduces the nuclear translocation of NF-κB p65 and proinflammatory cytokine (IL-6, TNF-a, and IL-1β) levels in the hippocampus, enhances cognitive function, improves learning and memory abilities, and exerts anti-neuroinflammatory effects in APP/PS1 transgenic mice [52]. The other mechanism related to memory enhancement is PDE4 inhibition, as PDE4 is a crucial component of N-methyl-D-aspartate (NMDA) receptor-mediated signal transduction pathway in memory processes [53]. In preclinical research, the NMDA receptor antagonist MK-801 has been shown to significantly increase the frequency of working and reference memory errors; however, pretreatment with rolipram effectively ameliorates these memory deficits [54]. Another potential mechanism through which PDE4 inhibition may exert its effects is in the stimulation of the extracellular signal-regulated kinase (ERK) pathway. NMDA receptors are known to regulate the ERK pathway, which is essential for oligodendrocyte development and memory mediation [55]. According to a study employing mice forced to undergo demyelination, rolipram partially improves oligodendrocyte progenitor cell (OPC) maturation through the MAPK route while raising ERK phosphorylation partially through the MEK-ERK pathway. Rolipram also inhibits TNF-α expression at mRNA and protein levels, downregulating IL-13, IL-5, and IFN-γ [56,57,58]. A recent study found that rolipram combined with a general PDE inhibitor pentoxifylline suppresses oxidative stress by significantly increasing the cAMP level, which is associated with increases in antioxidants, such as total thiol, a common antioxidant enzyme found in almost all living tissues that use oxygen catalase (CAT) and superoxide dismutase (SOD), in dorsal root ganglion (DRG) neurons. This study provides a new direction for further clinical investigations on the role of PDE4 inhibitors in aging-related neuropathy.

### 3.2. PDE4 and Its Selective Inhibitors in MDD

**Figure 1:**
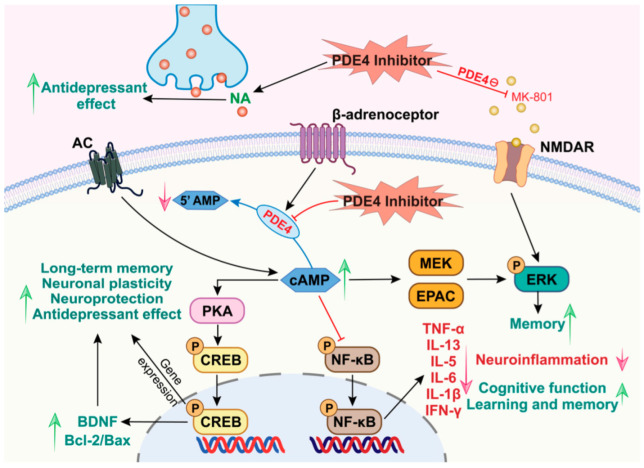
PDE4-dependent signaling pathway in Alzheimer’s disease and major depressive disorder.

Rolipram was initially investigated as an antidepressant due to its capacity to enhance the accumulation of cAMP triggered by neurotransmitter stimulation in the brain and increase neuron excitability for better cognition. This compound acts on both presynaptic and postsynaptic elements of monoaminergic transmission [59] and is characterized by a unique antidepressant mechanism that involves the stimulation of tyrosine hydroxylase activity. The mechanism is related to increases in neuronal activity and neurotransmitters by slowing dopamine degradation and increasing the synthesis and release of norepinephrine [60]. Furthermore, rolipram has demonstrated antidepressant-like and memory-enhancing effects in animal models [50,61]. The action of rolipram hypothesizes that it induces an increased rate of norepinephrine turnover along with the inhibition of cAMP metabolism, subsequently leading to adaptive changes in synapses [62]. Additionally, several studies have suggested that β-adrenoceptors play an important role in regulating PDE4 enzyme activity. An in vivo study identified PDE4 as the primary mediator of cAMP hydrolysis following the stimulation of β-adrenoceptors in the rat cerebral cortex. The PDE4A and PDE4B subtypes have been observed to be modulated by noradrenaline-mediated activity [63]. Consequently, the inhibition of PDE4 may have antidepressant-like effects due to changes in noradrenaline-mediated neurotransmission. cAMP is pivotal in relaying and amplifying signals received at cell surface receptors, playing essential roles in signal transduction cascades [64]. By degrading these cyclic nucleotides, PDEs can regulate the localization, duration, and amplitude of cyclic nucleotide signaling. Adenylate cyclase (AC) is synthesized when extracellular signals, such as a neurotransmitter or hormone, activate them. When cAMP is synthesized, it activates protein kinase A and phosphorylates other enzymes or transcription factors in the nucleus, such as CREB. It also controls cAMP-gated ion channels, causing a range of physiological responses depending on the specificities (Figure 1).

PDE4 is the primary mediator of cAMP hydrolysis (following the stimulation of β-adrenoceptors). PDE4 inhibition causes cAMP build-up in the brain and counteracts the effects of MK-801 on NMDA receptors (NMDAR). PDE4 inhibitors exert antidepressant-like effects by increasing norepinephrine levels. PDE4 inhibitors can reduce inflammation, promote neuroprotection, and improve memory/cognitive function by activating the cAMP/PKA/CREB, AC/cAMP/PKA, cAMP/MEK/ERK, cAMP/EPCA/ERK, cAMP/NF-κB, and NMDAR/ERK signaling pathways.

## 4. Roles of PDE4 Isoforms, PDE4A, PDE4B and PDE4D, in Cognitive Deficits and Psychiatric Disorders Associated with AD

PDE4 is classified into four subtypes: PDE4A, PDE4B, PDE4C, and PDE4D, each of which comprises 3 to 11 splice variants. These variants result from different truncations at the N-terminal and are classified into four categories based on the conservation of N-terminal regions: long-form, short-form, super short-form, and dead-short form [38]. The catalytic domains of all PDE4 isoforms (except PDE4A) contain an ERK phosphorylation site, which provides inhibitory or stimulatory regulation of PDE4 activity in a variant-specific manner. The distributions of PDE4 subtypes are brain-region specific, indicating differential roles of PDE4 subtypes in CNS functions [38]. PDE4A and PDE4D are predominantly expressed in the hippocampus, cerebral cortex, olfactory system, and brain stem, suggesting that these two subtypes may be important in the mediation of antidepressant activity and memory; PDE4B is primarily expressed in the basal ganglia and related areas such as the caudate putamen, nucleus accumbens, amygdala, and specific thalamic nuclei, indicating that PDE4B is primarily associated with affective properties and disorders, notably antidepressant effects, rather than pro-cognitive actions, PDE4D is generally associated with cognition; PDE4C is primarily found in peripheral tissues and is not abundantly expressed in the CNS [65,66,67,68] (Table 1).

Rolipram has illuminated the significance of PDE4 in the regulation of memory processes [81], but its practical utility has been limited due to the high rate of adverse effects [82]. Consequently, recent research has focused on developing drugs with improved PDE4 subtype selectivity to better understand their involvement in the CNS. Previous investigations have shown that the adverse effects of PDE4 inhibitors are more closely associated with PDE4D than with PDE4B, as evidenced by isoform-specific knockout animal studies [83,84,85]. In comparative analyses, the first PDE4B-selective inhibitor (Compound 33) has shown comparable efficacy to the PDE4D-selective inhibitor Cilomilast [86], yet exhibited significantly reduced emetic effects, even at doses 100 times higher. Furthermore, PDE4B knockout mice show a 90% reduction in TNF production compared to wild-type mice, although PDE4A and PDE4D knockout mice have normal TNF responses [87]. This study demonstrates that PDE4B could be a promising target for anti-inflammatory benefits while inducing fewer adverse effects than other PDE4 inhibitors. Most recent studies are paying more attention to developing isoform-specific knockout animal models to identify which PDE4 isoforms are most effective for various neuropsychiatric indications.

An in vitro study investigating the regulation of PDE4B and cAMP signaling has identified associations between the expression and subcellular localization of PDE4B in hippocampal neurons with long-term potentiation. This regulatory mechanism appears to play a crucial role in synaptic plasticity and cellular memory formation [88]. A complementary in vivo study utilizing mice with a catalytic domain mutant form of PDE4B (PDE4BY358C/Y358C) displayed a decreased ability to hydrolyze cAMP, resulting in decreased anxiety and increased exploration time in the open field test. Cognitive enhancements regarding learning and memory were also found, along with enhanced neurogenesis in PDE4B catalytic domain mutant mice [72]. Furthermore, these PDE4B knockout mice displayed reduced immobility in forced swimming tests [71], indicating a relationship with depression-like behavior. Additional research highlights a decrease in startle response during pre-pulse inhibition in PDE4B knockout mice, supporting the role of PDE4B in psychiatric diseases [83]. PDE4B inhibitors can potentially reduce neuroinflammation by blunting microglial cytokine production triggered by Aβ in the brain [89]. Over the past decades, numerous PDE4 inhibitors have been designed and synthesized, among which roflumilast, apremilast, and crisaborole were approved for treating inflammatory airway diseases, psoriatic arthritis, and atopic dermatitis, respectively [90]. In contrast, the dramatic efficacies of PDE4 inhibitors are often accompanied by severe adverse effects. Substantial advances should be made to mitigate the adverse effects and obtain a better benefit-to-risk ratio.

Recent investigations have demonstrated that PDE4D inhibitors enhance cognitive functions by upregulating the cAMP/PKA/CREB signaling pathway, which is crucial for memory consolidation [91]. In a study comparing the effects of PDE4B and PDE4D selective inhibitors, the PDE4B selective inhibitor A-33 showed an antidepressant-like profile with reduced immobility time in forced swimming and tail suspension tests and reduced latency to feed in the novelty-suppressed feeding test. Meanwhile, the PDE4D selective inhibitor D159687 exhibited clear cognitive-enhancing properties but without antidepressant- or anxiolytic-like benefit [74]. Additionally, the PDE4D selective inhibitor D159687, along with allosteric modulators D159404 and D159687, were found to be more potent than rolipram in the scopolamine-impaired Y-maze and novel object recognition behaviors, although the maximum cognitive benefits were not as expected [92]. A subsequent study evaluated the memory-enhancing effects of BPN14770, an allosteric inhibitor of PDE4D, on both wild-type and humanized PDE4D mice. The study concluded that the targeted inhibition of PDE4D through a single, acute dose of BPN14770 increased brain cAMP levels, enhanced extended phases of LTP, reversed scopolamine impairment of short-term memory, and improved long-term memory through a PKA-dependent mechanism [80]. Moreover, BPN14770 exhibited a 100-fold increase in efficacy for enhancing memory in both Y-maze and novel object recognition tests in humanized mice compared to wild-type mice, showing the potential for a significant role in memory [80]. Further research is underway to develop analogs or derivatives of BPN14770 that selectively target PDE4D, indicating their effectiveness in neurodevelopmental disorders and depressive-like behavior [85]. Increasing studies have shown that PDE4D knockout mice exhibit decreased immobility time in both tail suspension and forced swim tests compared to those of wild-type mice, which are similar to the behavior observed when the antidepressants desipramine and fluoxetine are administered [59]. These studies further support the crucial role of PDE4D in depression-like behavior. Additionally, a recent study demonstrated that the PDE4 inhibitor apremilast suppressed MAPK and PI3K-mTOR signaling by reducing the expression of chemokines and chemokine receptors in the colon, indicating that PDE4 plays a role in peripheral system disorders besides its role in CNS disorders [93]. Although it remains uncertain which PDE4 isoform is most effective on CNS disorders, the current evidence supports the fact that PDE4D is an essential mediator of rolipram’s antidepressant-like effects and that PDE4D-regulated signaling may play a key role in the neuroinflammation of MDD and ADRD [94].

## 5. Conclusions

AD and related depression and anxiety represent significant neuropsychiatric conditions that collectively affect millions of people worldwide. According to the current research, neuroinflammation plays a crucial role in both disorders’ development and exacerbation, and PDE4 is a promising pharmacological target for its diverse roles in the physiological and behavioral modulation of the CNS. The pharmacological inhibition of PDE4 has been confirmed to elevate cAMP levels and the related signal transduction pathways, attenuating inflammatory responses and enhancing neuroprotection. With a further understanding of the action of PDE4 and its isoforms in the CNS and advances in drug development technology, we are expected to develop more selective and effective PDE4 isoform-specific inhibitors for the treatment of neuropsychiatric disorders associated with AD.

## Figures and Tables

**Table 1:** Effects of different PDE4 subtype inhibitors on central nervous system function.

PDE4 Subtypes	Disease	Drug/Model	Result	References
PDE4A	Anxiety	PDE4A knockout mice	Enhanced emotional memory	Hansen et al., 2014 [69]
PDE4A	Sleep deprivation	pAAV_9_-CaMKIIα0.4-eGFP/pAAV_9_-CaMKIIα0.4-PDE4A5^catnull^-VSV	Improved memory consolidation	Havekes et al., 2016 [70]
PDE4B	Anxiety	PDE4B knockout mice	Attenuated anxiety-like behavior	Zhang et al., 2008 [71]
PDE4B	Cognitive dysfunction	PDE4B^Y358C/Y358C^ mice	Anxiolytic effects, facilitated memory acquisition, enhanced neurogenesis	McGirret al., 2016 [72]
PDE4B	Traumatic brain injury	A33	Anti-inflammatory effect, reduced neuronal loss	Wilson et al., 2017 [73]
PDE4B	Depression, anxiety	A33	Antidepressant-like effect	Zhang et al., 2017 [74]
PDE4B	Multiple sclerosis	A33	Anti-inflammatory effect	Schepers et al., 2022 [75]
PDE4B	Sleep deprivation	A33	Alleviates memory deficits	Zhao et al., 2024 [65]
PDE4B	AD	*App*^NL-G-F^/*Pde4b*^Y358C^ mice	Anti-inflammatory effect, protective effects on brain metabolism and spatial memory	Armstrong et al., 2024 [76]
PDE4B/4D	AD	Roflumilast	Improved learning and memory, attenuated depression-like behavior	Wang et al., 2020 [51]
PDE4D	/	PDE4D knockout mice	Increased learning ability and memory, nerve regeneration	Rutten et al., 2008 [77]
PDE4D	Depression and memory deficits	4DmiR (PDE4D knock-down mice)	Antidepressant-like effect, improved memory	Wang et al., 2015 [78]
PDE4D	Fragile-X syndrome	BPN14770	Improved dendritic spine morphology, social interaction, and natural behaviors	Gurney et al., 2017 [79]
PDE4D	Depression, anxiety	D159687	Improved cognitive	Zhang et al., 2017 [74]
PDE4D	AD	BPN14770	Improved cognitive and memory, neuroprotective and antiapoptotic effects	Wang et al., 2020 [80]
PDE4D	Multiple sclerosis	Gebr32a	Improved spatial memory and reduced visual evoked potential latency times	Schepers et al., 2022 [75]
PDE4D	Sleep deprivation	zatolmilast	Mitigated memory impairments	Zhao et al., 2024 [65]

## Data Availability

No new data were created or analyzed in this study. Data sharing is not applicable to this article.
